# Hardness and Wear Resistance of Dental Biomedical Nanomaterials in a Humid Environment with Non-Stationary Temperatures

**DOI:** 10.3390/ma13051255

**Published:** 2020-03-10

**Authors:** Daniel Pieniak, Agata Walczak, Mariusz Walczak, Krzysztof Przystupa, Agata M. Niewczas

**Affiliations:** 1 Department of Mechanics and Machine Building, University of Economics and Innovations in Lublin, Projektowa 4, 20-209 Lublin, Poland; daniel.pieniak@wsei.lublin.pl; 2 The Main School of Fire Service, Faculty of Safety Engineering and Civil Protection, Slowackiego 52/54, 01-629 Warsaw, Poland; awalczak@sgsp.edu.pl; 3 Department of Materials Engineering, Faculty of Mechanical Engineering, Lublin University of Technology, Nadbystrzycka 36, 20-618, Lublin, Poland; m.walczak@pollub.pl; 4 Department of Automation, Faculty of Mechanical Engineering, Lublin University of Technology, Nadbystrzycka 36, 20-618, Lublin, Poland; 5 Department of Conservative Dentistry with Endodontics, Medical University of Lublin, Karmelicka 7, 20-080 Lublin, Poland; agata.niewczas@umlub.pl

**Keywords:** microhardness, scratch resistance, sliding wear, thermocycling, dental biomaterials

## Abstract

This study discusses a quantitative fatigue evaluation of polymer–ceramic composites for dental restorations, i.e., commercial material (Filtek Z550) and experimental materials Ex-nano (G), Ex-flow (G). Their evaluation is based on the following descriptors: microhardness, scratch resistance, and sliding wear. In order to reflect factors of environmental degradation conditions, thermal fatigue was simulated with a special computer-controlled device performing algorithms of thermocycling. Specimens intended for the surface strength and wear tests underwent 10^4^ hydrothermal fatigue cycles. Thermocycling was preceded by aging, which meant immersing the specimens in artificial saliva at 37 °C for 30 days. Microhardness tests were performed with the Vickers hardness test method. The scratch test was done with a Rockwell diamond cone indenter. Sliding ball-on-disc friction tests were performed against an alumina ball in the presence of artificial saliva. A direct positive correlation was found between thermocycling fatigue and microhardness. The dominant mechanism of the wear of the experimental composites after thermocycling is the removal of fragments of the materials in the form of flakes from the friction surface (spalling). Hydrothermal fatigue is synergistic with mechanical fatigue.

## 1. Introduction

Dental restorative materials are classified as resin-based composites (RBCs) [1]. In the literature, they are also often listed as light-cured polymer matrix ceramic composites (LC PMCCs). Dental materials are used, because long-term degradation processes such as fatigue, wear, and caries often create a need to repair human teeth [2]. LC PMCCs are characterized by good performance. On the basis of the work [3], the failure of restorative composites may occur, among others, due to poor wear resistance.

The inorganic phase is of key importance for the performance of resin composites. Not only its contribution to the structure of the composite, but also its shape, type, and size are significant [4]. It determines usable properties, i.e., aesthetic, mechanical, density (consistency), and the purpose of the composite material. They depend on properties such as Vickers hardness, modulus of elasticity, work of force on deformation to failure (from bending tests), and the critical value of the stress intensity coefficient [5]. On the other hand, mechanical strength also depends on the filler. It has been found that the increase in the content of ceramic filler particles results in improved strength and this relationship is exponential [6,7,8,9]. Generally, the smaller the average particle size of the filler, the better the polishability and smoothness. Larger ceramic particles provide higher immediate mechanical resistance and lower material shrinkage. The level of shrinkage is also associated with the volume content of the filler in the composite structure [10]. In contrast, nanofillers have a positive effect on the tribological resistance of the RBCs outer surface layer.

Fillings and other dental applications made of RBCs are exposed to oral factors [11]. One such factor is the cyclical thermal load caused by the wear from beverages and foods at different temperatures. Some authors reported that within five years, 6000 cycles of thermal loads may occur in the oral cavity [12,13]. Another work reported that 2000 thermal cycles (TCs) occur for 200 days [14]. The thermal load may have the character of a thermal shock caused by sudden contact with a medium at a temperature that is several degrees Celsius lower or higher [15].

In fact, TCs play an important role in the durability of RBCs. The success of modern materials is defined by the durability of the application in the conditions of the environment, which is the oral cavity [16]. Tests under static conditions of one-cycle unconditioned materials do not correspond to clinical conditions [17]. Therefore, tests under cyclic loading should be carried out. The results of clinical trials do not show differences in the degradation of composites of different structure, with macro- and microfills, as well as hybrid and nanohybrid composites [18]. In addition, superficial observations, carried out in clinical settings, at incomplete periods of use, often lead to premature interpretations, mainly due to the dynamic behavior of stability curves [19,20,21]. However, in laboratory tests of the fatigue process, you can focus on individual degradation mechanisms. For this purpose, the thermocycling method was developed. Thermocycling is a process of simulation of temperature fluctuations modeling the conditions of eating, drinking, and breathing, among other physiological states [22,23,24,25]. However, it should be emphasized that the results obtained with the use of laboratory thermocycling methods are imperfect. In real conditions, there are significant individual differences [26]. People have different habits regarding eating, drinking, and taking care of the mouth. It is not possible to simulate everyone’s routine. However, the laboratory thermocycling method gives good approximations.

RBCs fatigue degradation was the subject of research undertaken in [23,27,28,29,30]. In [27], the influence of TCs on wear resistances of LC PMCCs was demonstrated. The authors found that the softening of polymer surfaces may be important, depending on the presence of water, the temperature of the polymer and the difference between the glass transition temperature and the operating temperature. In the studies, researchers usually evaluated the influence of TCs on composites strength and Young’s modulus. Currently, there are not many papers focusing on the impact of TCs on contact strength and resistance to tribological wear [31,32,33,34]. It should be emphasized that the durability of RBCs due to thermocycling varies and depends on many changing factors.

Despite the continuous development of resin composites, the wear process of surface due to abrasion is still the main factor in causing clinical failures and premature replacement of teeth reconstruction [34]. The increasing number of new RBCs forces the need for long-term in vivo wear, and it is still crucial to prove it in reliable in vitro testing [35]. The wear process is complex, and it is the result of a combination of sliding friction and three-body abrasion [36,37,38], which occur not only in the physiological process of chewing, but also in pathological bruxism. The rate of abrasive wear can be affected by TCs [12]. In [32], the influence of thermocycling on tribological properties depending on the structures of resin composites was demonstrated.

The aim of this work was to evaluate the effect of thermocycles on sliding wear resistance of experimental and commercial RBCs for dental restorations. Hardness and scratch tests were performed to investigate the durability and the relationship between durability and sliding wear resistance.

## 2. Materials and Test Methods

### 2.1. Materials

Three LC PMCCs were selected for testing: one commercial composite (Filtek Z550) and two experimental composites (Ex-nano (G) and Ex-flow (G). Filtek Z550 is a reference material used in dental composites research, which contains a nanoparticle filler. The parameters of this material are described in Table 1. Filtek Z500 is classified as a nanohybrid composite. The material includes inorganic filler particles at a nanoscale. Experimental materials were given to the studies by one of dental composites manufacturers in order to determine the mechanical properties of new composites in oral environment conditions. Experimental materials consisted of particles at a nanoscale, and they had different filler contents. The dispersion of the filler particles in the matrix (in all tested composites) was random.

Specimens were prepared in accordance with the manufacturers’ recommendations. This meant that the specimens were shaped by a single operator in a metal split mold and then light-cured using Megalux LED (Megadenta, Radeberg, Germany) at 1200 mW/cm^2^ for 40 s with a soft start system. After photopolymerization, specimens were polished with abrasive discs (granulation P600, P1200, and P2400) on a single wheel grinder and polisher Saphir 550 (ATM Gmbh, Mammelzen, Germany) equipment and then cleaned in water. Wear truck images were made by a Phenom G2 Pro scanning electron microscope (Phenom-World BV, Eindhoven, Netherlands). The microscopic structure of the tested materials is presented in the paper [11].

### 2.2. Method of Thermocycling

All of the tested specimens were immersed in artificial saliva at 37 °C for 30 days to simulate the aging process. Then, half of them underwent microhardness, microscratch, and wear sliding tests, and the second half underwent thermocycling. In this study, groups were formed according to the type of material as the aging procedure only (0 TC) and the aging and thermocycling procedure. All thermocycles were conducted between 5 and 55 °C for a dwell time of 30 s. The time of filling and emptying the vessel with a working liquid (water) was 15 s. The number of TCs was 10^4^, which according to the review article [39] was considered to be a sufficient number.

### 2.3. Microhardness Testing

Microhardness testing is a method used for indirect assessment of some clinically important properties of composites [40,41,42,43,44]. Microhardness tests were conducted using disc-shaped specimens, according to the ISO4049 standard. The test was performed using the Vickers hardness test method. In this method, a square-based pyramid diamond indenter with a face angle of 136° was impressed onto the surface of the test specimen. The tests were conducted using a Futertech FM 700 (Future-Tech Corp., Kawasaki-City, Japan) apparatus. The load used was 50 gf (grams of force), and the penetration time was 20 s. Measuring coordinates were set to cover the entire surface area of a specimen and were the same for all specimens. Two surfaces, exposed to curing light (LC) and not exposed to curing light (NLC), were tested.

### 2.4. Microscratch Tests

**Figure 1:**
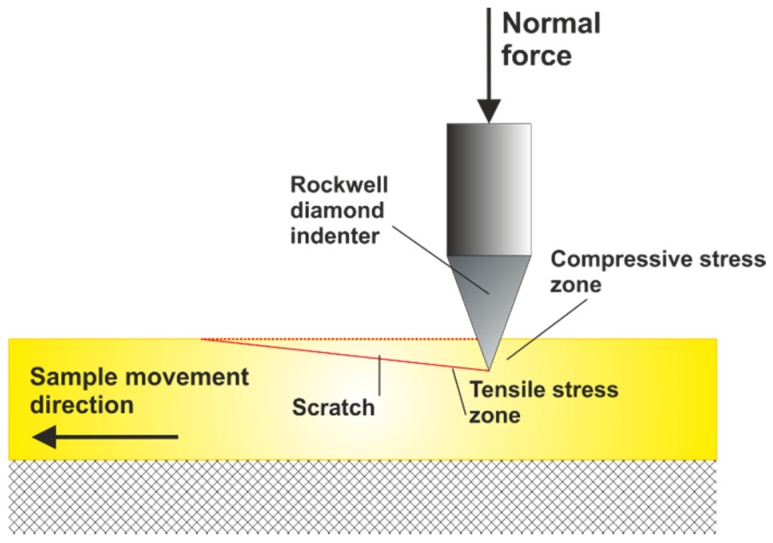
Schematic of the microscratch test.

Scratch tests were conducted using a Micro Scratch Tester (MST) (Anton Paar GmbH, Ostfildern, Germany), and a Rockwell diamond cone stylus was used. The indenter was incrementally loaded with loads in the range from 0.5 to 5 N and moved along a length of 0.5 mm. A schematic presentation of the test is shown in Figure 1.

### 2.5. Wear Sliding Test

**Figure 2:**
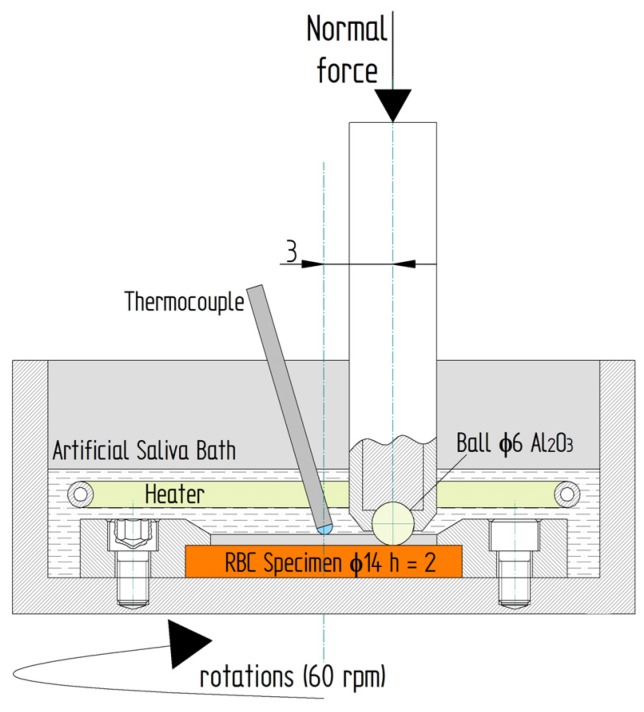
A schematic of the tribological test.

Sliding wear tests are used in biomaterial testing [45,46,47]. Dental biomaterials are also tested in this way [2,3]. Wear sliding resistance tests of dental composites were conducted using a microtribometer (CSM Instruments SA, Peseux, Switzerland) (Figure 2). The parameters of the test are shown in Table 2.

Reference and conditioned specimens were tested using the ball-on-disk method (Figure 2) in artificial saliva at a constant temperature of 37 °C. The test frequency was 1 Hz. This frequency is considered compatible with the physiological process of chewing and was reported by other researchers [37,48,49,50]. The number of specimens in each group was 5 (N = 5). There are various wear measurement methods. Some measure weight loss [51], while others determine volumetric wear [52,53]. In the present study, volumetric wear was measured using a Dektak 150 surface profilometer (Veeco, Plainview, USA, NY, USA). All tested specimens were measured on the transverse plane to the sliding direction circle. The distance between profiles was about 35 degrees, and 10 measurements were made around the perimeter of the wear scar.

In order to compare the mean values in the groups of sliding wear test results, a one-way analysis of variance (ANOVA) was made. The hypothesis was that the mean values in the groups were different for *p* ≤ 0.05.

## 3. Results and Discussion

### 3.1. Microhardness

**Figure 3:**
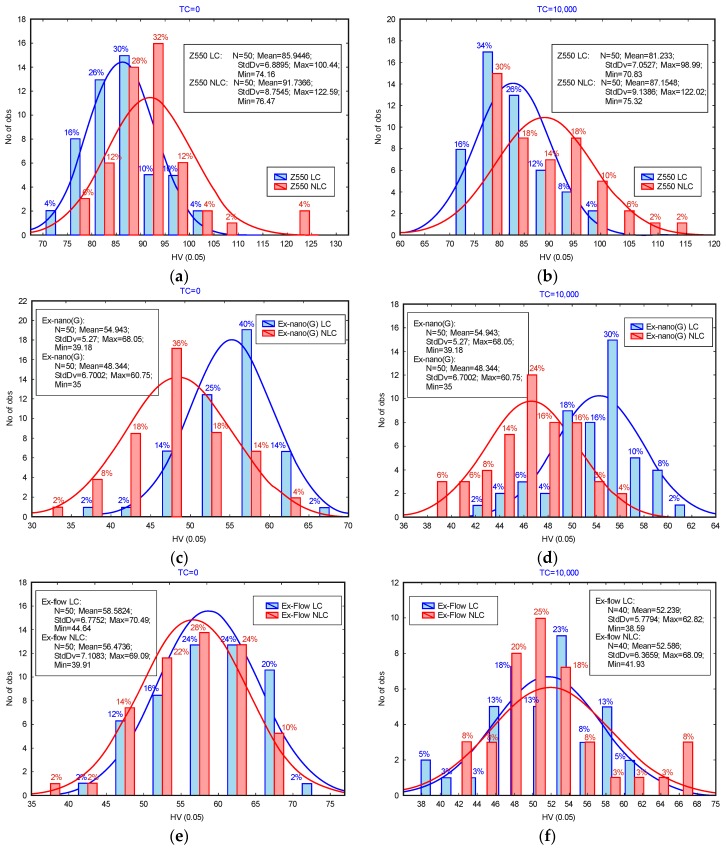
Frequency distributions of microhardness test results for: (**a**) Z550, (**b**) Z550 after thermal fatigue, (**c**) Ex-nano (G), (**d**) Ex-nano (G) after thermal fatigue, **(e**) Ex-flow (G), and (**f**) Ex-flow (G) after thermal fatigue.

Hardness assessment is widely used by RBCs researchers. The microhardness test allows for the assessment of the mechanical properties of the composite surface [54]. In addition, there are strong correlations between the composite microhardness and elastic modulus values, photopolymerization depth, and the level of polymerization shrinkage, and the relationship between the composite microhardness and the level of polymerization shrinkage is strongest among these three [40]. In Figure 3, the frequency distributions of the results of microhardness tests are presented. The graphs compare the results obtained for surfaces LC and NLC. The graphs for the reference specimens (0 TC) and specimens subjected to thermal fatigue (10,000 TCs) are displayed side by side for comparison.

Concerning restorative resin, the composite Z550 was characterized with the highest microhardness. The microhardness values of the experimental composites were similar, and the microhardness of the composite Ex-flow (G) was slightly higher than that of the composite Ex-nano (G). Hydrothermal degradation phenomena of microhardness for the test specimens were similar.

In the tests presented in this work, slight decreases in microhardness were observed after loading the samples with TCs. The decrease in microhardness of materials subjected to cyclic hydrothermal loads was noted [55,56] (temperature range: 5–55 °C, number of cycles: 1 × 10^4^). However, many studies have noticed an improvement in the surface properties of RBCs under the influence of cyclic thermal loads. For example, in [57], an increase in the hardness of a material called Sinfony was recorded under the influence of hydrothermal loads (temperature range: 5–55 °C, number of cycles: 5 × 10^3^). The authors [58] observed an increase in the nanoindentant hardness of composites as a result of hydrothermal cycles (temperature range: 5–55 °C, number of cycles: 2 × 10^3^) and immersion of materials for 48 h in a liquid at 55 ± 5 °C.

### 3.2. Scratch Tests

In the research presented in this work, a Rockwell indenter was used. According to [36], unlike using a spherical indenter, the undoubted advantage of testing with a sharp indenter, such as a Rockwell indenter, is a good chance of contact of the indenter with at least some nanofiller particles. In this case, the Hertz contact surface is only about 5 times larger than the average particle size of the filler.

**Figure 4:**
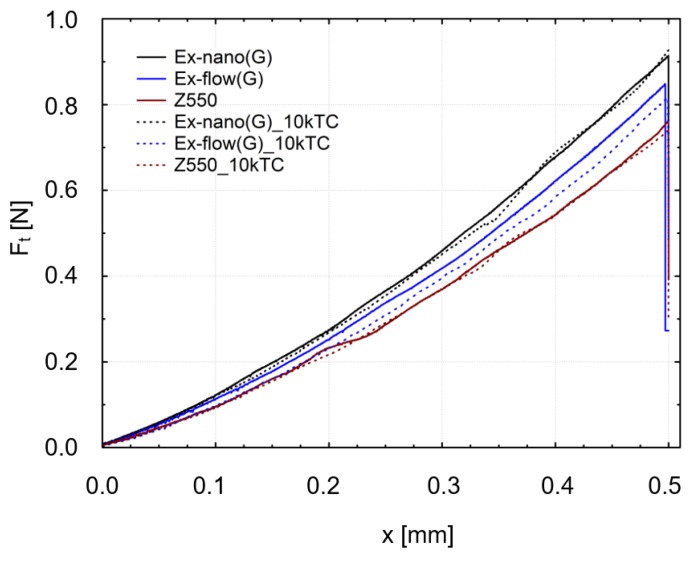
Mean curves of the friction force (F_t_) as a function of the indenter position (x) (10,000 thermal cycles (TCs)).

**Figure 5:**
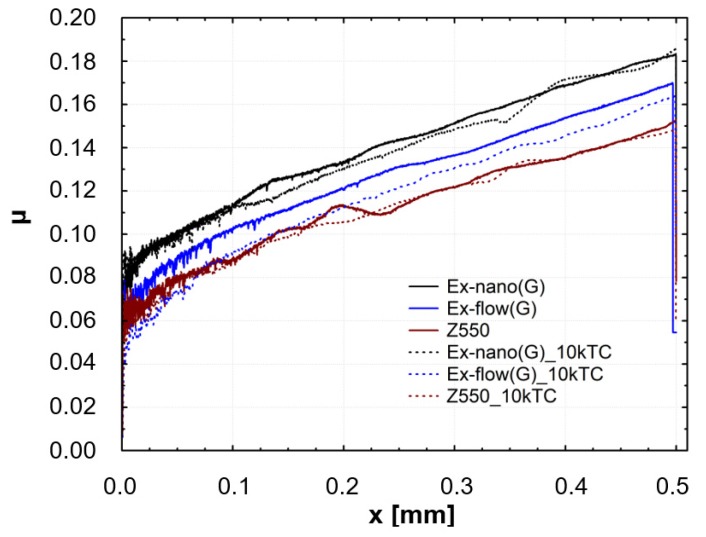
Mean curves of the coefficient of friction (μ) as a function of the indenter position (x).

**Figure 6:**
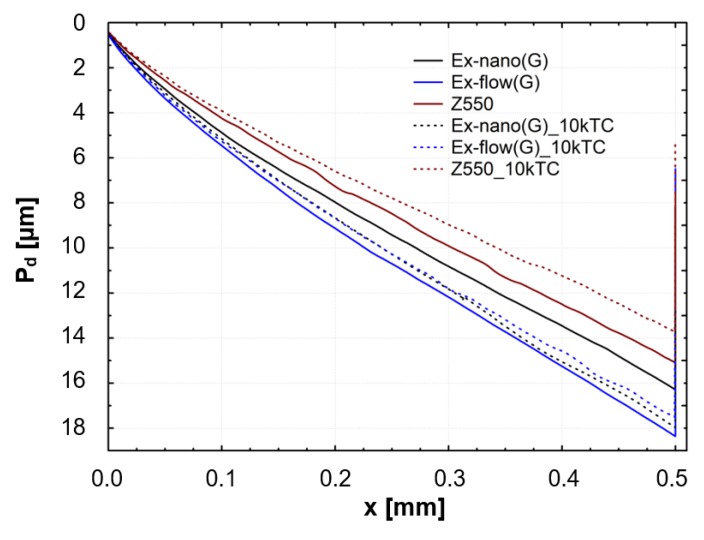
Mean curves of the indenter penetration depth (P_d_) as a function of the indenter position (x).

**Figure 7:**
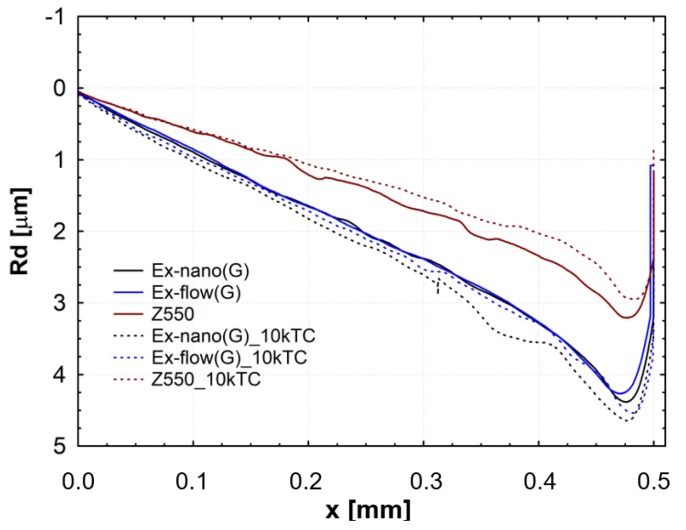
Mean curves of the indenter residual depth (R_d_) as a function of the indenter position (x).

**Figure 8:**
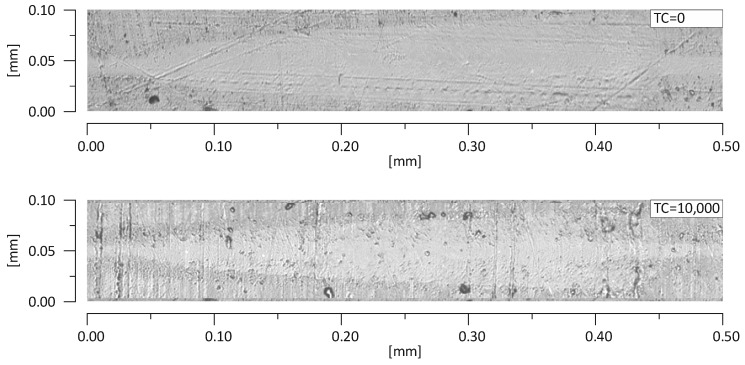
Scratches on the surfaces of the test specimens Z550 with aging only (0 TC) and after thermal fatigue (10,000 TCs).

**Figure 9:**
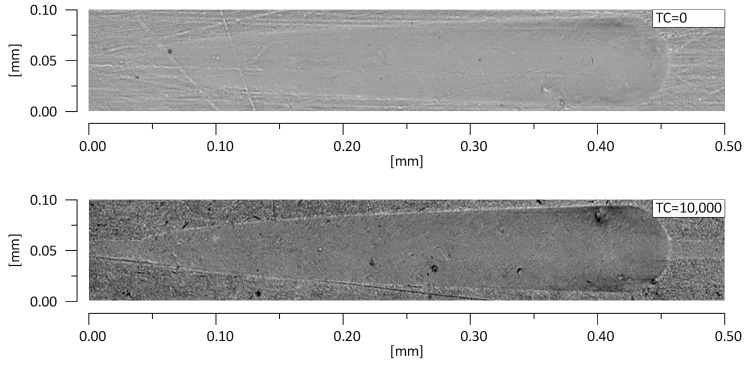
Scratches on the surfaces of the test specimen Ex-nano (G) with aging only (0 TC) and after thermal fatigue (10,000 TCs).

Results of scratch tests of the investigated materials are shown. The graphs show the mean curves of measurements made on the light-cured surfaces of specimens after aging only (0 TC) and after thermal fatigue cycling (10,000 TC). Mean curves of the friction force (F_t_) as a function of the indenter position (x) are presented in Figure 4. Mean curves of the coefficient of friction (μ) as a function of the indenter position (x) are shown in Figure 5. Mean curves of the depth of indenter penetration (P_d_) as a function of the indenter position (x) are displayed in Figure 6. Mean curves of the residual depth (R_d_) as a function of the indenter position (x) are shown in Figure 7. In addition, selected images of scratches obtained using an optical microscope are shown in Figure 8 and Figure 9.

Referring to the test results obtained, the composite Z550 showed the highest resistance of scratch failures expressed in the residual depth of scars. The mean residual depth of scars was slightly smaller after thermocycles. In the case of experimental composites, the residual depths of scars were similar and slightly higher after thermocycles. Similar relationships were found for penetration depth of the indenter (P_d_), and the scar depth for the material Z550 was the lowest in the case of Z550 after thermocycles. To supplement the quantitative analysis, Figure 8 and Figure 9 summarize the scratches obtained on the surfaces of the reference materials Z550 and the experimental material Ex-nano (G). In the comparison of representative scratches on the surfaces of all tested materials, 0 TC referred to samples that underwent aging only, and the use of 10,000 TCs was for specimens after thermocyling. The qualitative assessment confirmed that permanent scratching on the surface of the experimental material Ex-nano (G) was more extensive. The comparison of Figure 5 and Figure 7 showed that the values of the R_d_ parameter were directly proportional to the values of the parameter μ for the same material. The lowest mean residual depth of scars R_d_ was shown for the material Z550, and the friction coefficient μ values were also the lowest for this material. Similar relationships were demonstrated for the experimental materials Ex-nano (G) and Ex-flow (G).

The dominating process of oral RBCs wear is abrasion. In the oral cavity, the wear process is particularly intense when using a favorable diet, e.g., eating large amounts of hard bread. Abrasive wear may be the result of three-body wear [59]. This type of cooperation arises as a result of redistribution of normal and tangential occlusal forces through an intermediary layer, e.g., chewing food (more precisely, suspension of food particles and wear products [37]), to all bodies of friction association [60]. It has also been noticed that the process of this type of wear is divided into an early phase and a late phase. In the early phase, large pieces of food are between an opposing teeth and the redistribution of forces, resulting in a different stress distribution. In the late phase, the surfaces of the opposing teeth approach each other, and a mixed friction may occur. The suspended particles are transferred in microcavities of the composite surface. In subsequent stages, they may cause the scratching of the opposing surfaces [37]. In addition, wear in this phase can be the most intense, making scratch resistance an important usable parameter of a dental composite.

### 3.3. Sliding Wear

**Figure 10:**
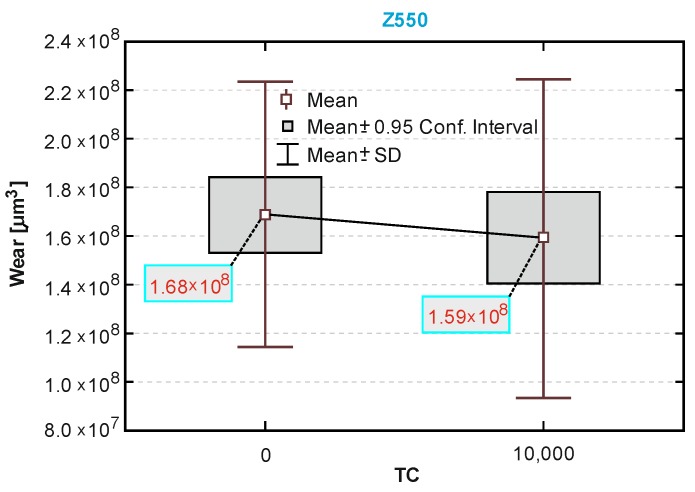
A box-and-whiskers plot of the wear resistance of the composite Z550.

**Figure 11:**
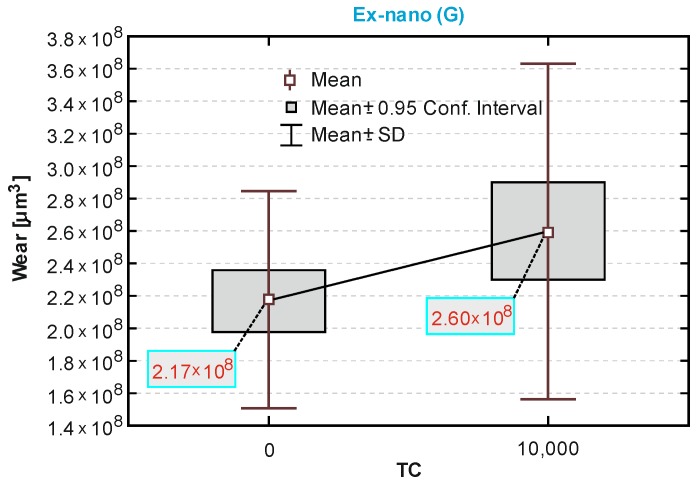
A box-and-whiskers plot of the wear resistance of the N-fill-flow composite.

**Figure 12:**
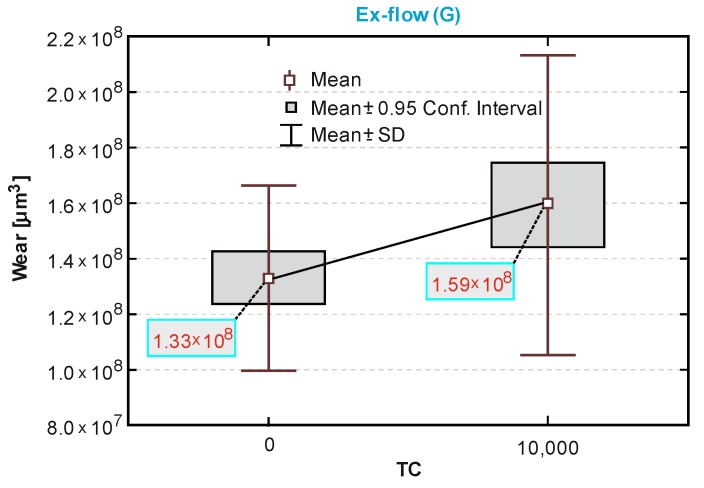
A box-and-whiskers plot of the wear resistance of the N-fill-flow composite.

The box-and-whiskers plots in Figure 10, Figure 11 and Figure 12 showed a statistical interpretation of the results of the wear resistance (tribological) tests. The diagrams showed the mean, confidence levels (±0.95) and standard deviations. The number of TCs is plotted on the x-axis. In one-way ANOVA analysis, a significant difference was proved between the results of sliding wear tests of the Ex-nano (G) with aging only and after thermocycles (*p* = 0.0166). Statistically significant differences were also shown for the composite Ex-flow (G) (*p* = 0.0045). The group results of sliding wear tests of the material Z550 with aging only and after thermocycling did not differ statistically significantly (*p* = 0.4363). This is consistent with the relationships shown in Figure 10, Figure 11 and Figure 12.

The volumetric wear after aging only was the highest for the experimental material Ex-nano (G) and the lowest for the experimental material Ex-flow (G). Lower sliding wear values of flow-type composites compared to those of universal composites were also observed in the work [11]. The average volumetric wear of Z550 after thermocycling was slightly lower (0.09 × 10^8^ µm^3^ lower) than the surface wear of samples subjected to aging only. The mean volumetric wear of the material Ex-nano (G) was 0.43 × 10^8^ µm^3^ higher after thermocycling, and the material Ex-flow (G) was 0.26 × 10^8^ µm^3^ higher after thermocycling. The presented research results were varied, and also the structures of the tested composites were different. In previous works [1,61,62], it was proved that the wear of RBCs depends on the content of the filler in the matrix and its size. Generally, it can be assumed that the participation of nanoparticles in the structure of a surface layer causes strengthening and homogeneity as well as slowing down the wear process [34,63]. In addition, nanofiller particles can have a positive effect on load transfer from the matrix to the filler [19]. With a smaller filler size, the surface area-to-volume ratio is greater, which leads to better interaction, also by force, of the matrix filler [40]. In the present study, all composites contained a nanofiller. However, the degree of volumetric wear varied. In addition, the contact strength of the composites tested varied, which may have an indirect effect on tribological wear. It has been proven that wear resistance correlates strongly with the stress intensity factor (*K_IC_*) [64]. In [65], it was shown that the wear depends on the critical value of the square of the stress intensity coefficient (*K_1C_*^2^) and the hardness. More specifically, Fredrich [65] showed that the ratio of the volumetric wear work and the work done to overcome the friction is directly proportional to the ratio of the stress intensity coefficient and the friction coefficient. The hardness values of the tested experimental materials were similar, and that of the material Z550 was significantly higher. The friction work is proportional to the friction force, and the path of friction in the subject tests was the same for all samples. The work of friction is essentially the work of friction on the friction path. The average values of the friction work of the tested materials are presented in Table 3. The material Z550 was characterized by the highest friction work value. The mean values of the friction work for the material Z550 with aging only and after thermocycling were similar. A similar relationship was demonstrated for the material Ex-nano (G). However, in the case of the material Ex-flow (G), the average values of friction work differed more. As already mentioned, the friction work is calculated on the basis of the variable—friction force. Differences in the friction work of the material Ex-flow (G) with aging only and after thermocycling shown in the sliding wear test were also noticeable in the scratch test. In Figure 4, the average friction force curves do not coincide only for Ex-flow (G). In addition, the opposite is true for the parameter R_d_. The mean curves of the average depths of permanent scratching R_d_ of the material Ex-flow with aging only and after thermocycling coincide to the greatest extent (Figure 7), as do the average P_d_ waveforms (Figure 6).

**Figure 13:**
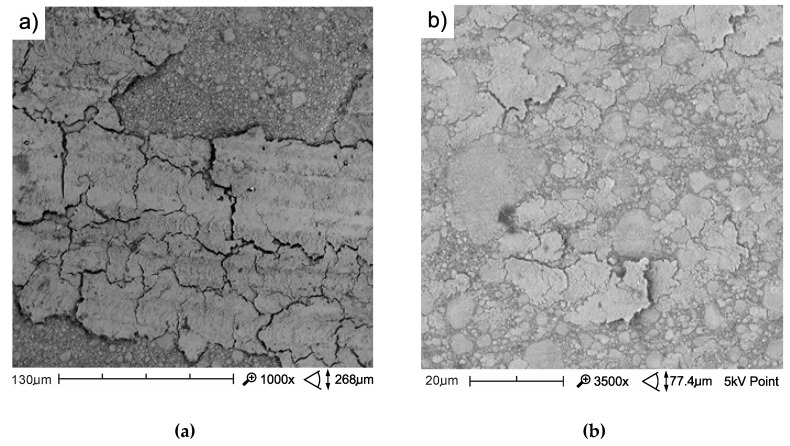
SEM image of a wear track of the composite Z550 with aging only in artificial saliva (AS) at 37 °C (**a**) and after 10^4^ TCs (**b**).

**Figure 14:**
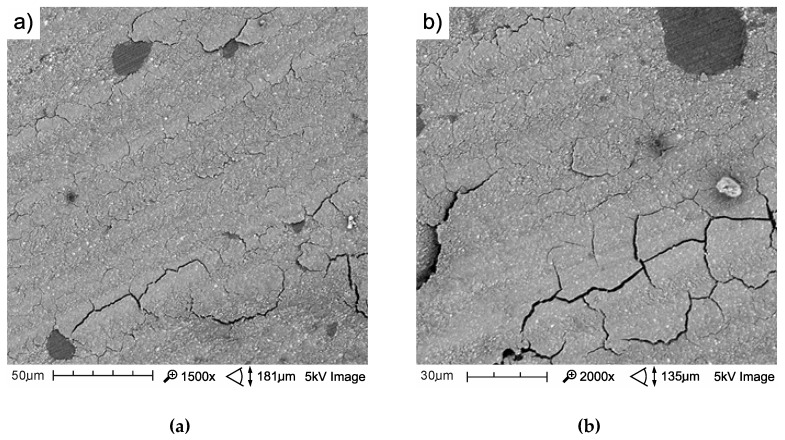
SEM image of a wear track of the composite Ex-nano (G) with aging only in AS at 37 °C (**a**) and after 10^4^ TCs (**b**).

**Figure 15:**
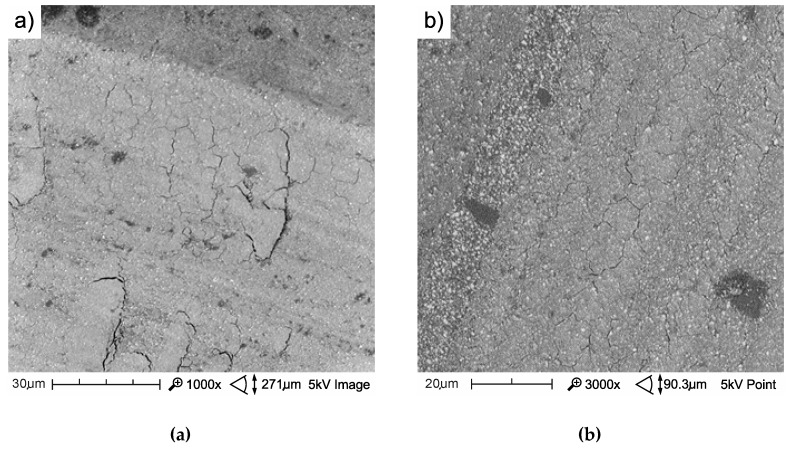
SEM images of a wear track of the composite Ex-flow (G) with aging only in AS at 37 °C (**a**) and after 10^4^ TCs (**b**).

The mechanism of friction surface destruction may be of significance for tribological wear. The mechanisms of the wear of the tested materials are not identical, and the effects of thermocycling on wear resistance were also varied. The SEM image of tribological wear is shown in Figure 13, Figure 14 and Figure 15. The following items show the tribological wear of Z550 (Figure 13), Ex-nano(G) (Figure 14) and Ex-flow (G) (Figure 15) sequentially. In the present study, it was noticed that hydrothermal fatigue caused a decrease in the fracture toughness of the surface layer. This condition may be indicated by damages in the form of flakes of the materials. 

In the case of the material Z550, the inverse relationship was observed after thermocycles. A smaller number of flakes characterized by spalling damage were observed, and flakes formed after TCs were smaller and more difficult to notice even at higher magnification on a scanning electron microscope (Figure 13). After TCs in the case of Z550, furrow wear dominated. It is possible that this wear mechanism translates into wear expressed quantitatively, and the wear resistance of Z550 material, expressed as the volumetric loss of a material on the friction surface, almost did not change after TCs (Figure 10).

In the case of Ex-nano(G) damage visible in Figure 14b, where the path of the material Ex-nano (G) after thermocycles is presented, was more extensive. There was damage that caused the formation of wear products in the form of flakes, which is described in tribology as spalling. The formation of wear products was gradual. The following stages of particle formation are listed as following: nucleation of microdefects, coalescence of microdeffects into a microgap, propagation of the crack gap, up to the exit to friction surfaces, detachment of wear products in the form of plates (flakes) [66]. In [36], it was found that delamination is associated with the formation of wear products in the form of patches and the main mechanism of flake formation is subsurface microcracks developing as a result of shearing in the soft polymer phase. The size of wear products in the form of patches depends on the propagation of subsurface cracks, which in turn is related to the level of normal and tangential loads to the surface [36]. In addition, in [14], the process of the formation of RBCs surface wear products was described.

It seemed that the spalling material Ex-nano (G) after thermocycles was facilitated in relation to the material Ex-nano (G) after aging only. In addition, the wear expressed quantitatively in the frame-mustache chart indicated a decrease in wear resistance of this material after TCs.

The wear of the material Ex-flow (G) after aging only in artificial saliva was the lowest, compared to those of the other tested materials. The wear of Ex-flow (G) after thermocycles was very similar to the wear of the reference material Z550. The average volumetric wear values were the same for two decimal places (Figure 10 and Figure 12), while the average wear values expressed as the area of the scar cross-section for Z550 and Ex-flow (G) were 8449.02 and 8457.92 μm^2^, respectively. Like Z550, the Ex-flow (G) susceptibility to the flake wear after thermocycles did not increased. Microcracks referred to in the paper [65] as cross notching, leading to the formation of flakes, were not very well developed (Figure 15). It is possible that cross notching is caused by mechanical fatigue. Moreover, mechanical fatigue was pointed out by researchers as one of the dominant wear mechanisms. Mechanical fatigue and abrasion [7], mechanical fatigue [67,68], microcutting and microcracking abrasion [38], and delamination [69] were indicated as the dominant mechanism of PMCCs wear. Mechanical fatigue leads to the formation of flakes, and progressive fatigue leads to their falling off from the friction surface. For polymeric structures, fatigue wear is caused by cracking of macromolecule chains. In addition, subsurface damage due to material discontinuity around the reinforcement may occur in reinforced plastics [70]. This discontinuity, as it is assumed, is the result of the mechanism of internal forces transfer by loaded filler particles towards a less hard matrix [19]. In addition, in [71], it was found that the delamination of composite phases is the dominant mechanism for brittle materials (and such are highly filled PMCCs) in the case of relatively high normal loads, which also occur in physiological processes [72].

## 4. Conclusions

Based on the in vitro studies presented—the effects of hydrothermal fatigue on microhardness and tribological wear of RBCs, the main conclusions are as follows:

- A direct positive correlation was found between thermocycling fatigue and microhardness. Decreases in microhardness were not high after the implementation of 10^4^ thermocycles.
- The dominant mechanism of the wear of experimental composites after thermocycling is the removal of fragments of materials in the form of flakes from the friction surface (spalling). Total wear was a combination of abrasive and fatigue wear, which resulted in flake extraction.
- The results presented in this paper indicated that in the case of the experimental materials, in particular Ex-nano (G), hydrothermal fatigue was synergistic with mechanical fatigue. In addition, thermal cycler loading can lead to reduced resistance to tribological wear.
- The results obtained did not indicate a correlation of Vickers hardness with sliding wear but indicated a correlation between Vickers hardness and scratch resistance.
- The SEM analysis of traces after tribological tests indicated that the nature of wear in both cases is quasi-brittle and is typical of materials with moderate toughness and yield strength and the damage zone has numerous microcrackings.

## Figures and Tables

**Table 1:** Details about the tested composites.

Parameter	Name of materials		
	Filtek Z550 (abbreviation: Z550)	Ex-nano (G)	Ex-flow (G)
**Manufacturer**	3M ESPE (USA)	–	–
**Composite type**	Nanohybrid composite	Nanocomposite	Semiliquid composite
**Matrix (resin)**	BIS-GMA, UDMA, BIS-EMA, PEGDMA, TEGDMA	BIS-GMA, UDMA, TEGDMA	BIS-GMA, UDMA, TEGDMA
**Filler type**	SiO_2_ (size: 20 nm), ZrO_2_/SiO_2_ nanoparticles (size: 5–20 nm) and ZrO_2_/SiO_2_ clusters (size: 0.6–1.4 mm)	The inorganic filler particles consist of barium aluminum, bore glass, highly dispersed silicon dioxide, and nanoparticles.	The inorganic filler particles comprise silica, dental glass (strontium aluminum boronsilicate glass), and nanoparticles.
**Filler content (wt %)**	82%	82%	74%

**Table 2:** Parameters of the sliding wear test.

Parameter	Characteristic value	Unit
**Radius**	3	mm
**Linear speed (rotation speed)**	1.88 (60)	cm/s (rev/min)
**Load**	5	N
**Distance travelled**	300	m
**Counterspecimen**	Al_2_O_3_ ball (diameter: 6 mm)	-
**Temperature**	37	°C
**Medium**	artificial saliva	

**Table 3:** Average values of the friction work of the tested materials.

Material	Average Values of the Friction Work (J)	
	After Aging Only (0 TC)	After Thermal Fatigue Cycling (10,000 TCs)
**Filtek Z550**	870.9	875.7
**Ex-nano(G)**	695.7	694.2
**Ex-flow(G)**	679.8	731.4

## References

[1] Finlay N., Hahnel S., Dowling A.H., Fleming G.J.P. (2013). The in vitro wear behavior of experimental resin-based composites derived from a commercial formulation. Dent. Mat..

[2] Kruzic J.J., Arsecularatne J.A., Tanaka C.B., Hoffman M.J., Cesar P.F. (2018). Recent advances in understanding the fatigue and wear behavior of dental composites and ceramics. J. Mech. Behav. Biomed. Mater..

[3] Chadda H., Satapathy B.K., Patnaik A., Ray A.R. (2017). Mechanistic interpretations of fracture toughness and correlations to wear behavior of hydroxyapatite and silica/hydroxyapatite filled bis-GMA/TEGDMA micro/hybrid dental restorative composites. Compos. Pt. B-Eng..

[4] Walczak A., Niewczas A., Pieniak D., Gil L., Kozłowski E., Kordos P. (2018). Temporary Stability of Compressive Strength of Flow and Universal Type LC PMCCS Materials. Adv. Mater. Sci..

[5] Antunes P.V., Ramahlo A., Carrihlo E.V.P. (2014). Mechanical and wear behaviours of nano and microfilled polymeric composite: Effect of filler fraction and size. Mater. Des..

[6] Lohbauer U., Belli R., Ferracene J.L. (2013). Factors involved in mechanical fatigue degradation of dental resin composites. J. Dent. Res..

[7] Braem M., Lambrechts P., van Doren V., Vanherle G. (1986). In vivo evaluation of four posterior composites: Quantitative wear measurements and clinical behaviour. Dent. Mater..

[8] Gonçalves F., Kawano Y., Braga R.R. (2010). Contraction stress related to composite inorganic content. Dent. Mater..

[9] Kim K.H., Ong J.L., Okuno O. (2002). The effect of filler loading and morphology on the mechanical properties of contemporary composites. J. Prosthet. Dent..

[10] Shalaby W.S., Salz U. (2007). Polymers for Dental and Orthopedic Applications.

[11] Pieniak D., Przystupa K., Walczak A., Niewczas A.M., Krzyzak A., Bartnik G., Gil L., Lonkwic P. (2019). Hydro-Thermal Fatigue of Polymer Matrix Composite Biomaterials. Materials.

[12] Leibrock H., Degenhart M., Behr M., Rosentritt M., Handel G. (1999). In vitro study on the effect of thermo- and load-cycling on the bond strength of porcelain repair systems. J. Oral Rehabil..

[13] Fischer J., Zbaren C., Stawarczyk B., Hammerle C.H.F. (2009). The effect of thermal cycling on metal–ceramic bond strength. J. Dent..

[14] Arsecularatne J.A., Chung N.R., Hoffman M. (2016). An in vitro study of the wear behaviour of dental composites. Biosurf. Biotribol..

[15] Makarian K., Santhanam S., Wing Z.N. (2018). Thermal shock resistance of refractory composites with Zirconia and Silicon-Carbide inclusions and alumina binder. Ceram. Int..

[16] Freeman R., Varanasi S., Meyers I.A., Symons A.L. (2012). Effect of air abrasion and thermocycling on resin adaptation and shear bond strength to dentin for an etch-and-rinse and selfetch resin adhesive. Dent. Mater. J..

[17] Drummond J.L. (2008). Degradation, fatigue and failure of resin dental composite materials. J. Dent. Res..

[18] Heintze S.D., Rousson V. (2012). Clinical effectiveness of direct class II restorations a meta-analysis. J. Adhes Dent..

[19] Bayne S.C. (2012). Correlation of clinical performance with “in vitro tests” of restorative dental materials that use polymer-based matrices. Dent. Mater..

[20] Przystupa K. (2019). Reliability assessment method of device under incomplete observation of failure. Proceedings of the 2018 18th International Conference on Mechatronics-Mechatronika (ME).

[21] Przystupa K. (2019). The methods analysis of hazards and product defects in food processing. Czech J. Food Sci..

[22] Akin H., Ozdemir A.K. (2013). Effect of corrosive environments and thermocycling on the attractive force of four types of dental magnetic attachments. J. Den. Sci..

[23] Morresi A.L., D’Amario M., Capogreco M., Gatto R., Marzo G.R., D’arcangelo C., Monaco A. (2014). Thermal cycling for restorative materials: Does a standardized protocol exist in laboratory testing? A literature review. J. Mech. Behav. Biomed. Mater..

[24] Vasiliu R.-D., Porojan S.D., Bîrdeanu M.I., Porojan L. (2020). Effect of Thermocycling, Surface Treatments and Microstructure on the Optical Properties and Roughness of CAD-CAM and Heat-Pressed Glass Ceramics. Materials.

[25] Wang J., Kochan O., Przystupa K., Su J. (2019). Information-measuring System to Study the Thermocouple with Controlled Temperature Field. Meas. Sci. Rev..

[26] Cavalcanti A.N., Mitsui F.H.O., Ambrosano G.M.B., Marchi G.M. (2007). Influence of adhesive systems and flowable composite lining on bond strength of class II restorations submitted to thermal and mechanical stresses. J. Biomed. Mater. Res. Part. B Appl. Biomater..

[27] Xu H., Eichmiller F., Smith D., Schumacher G., Giuseppetti A., Antonucci J. (2002). Effect of thermal cycling of whiskers - reinforced dental resin composites. J. Mater. Sci.-Mater. Med..

[28] Javaheri M., Seifi S.M., Aghazadeh Mohandesi J., Shafie F., Lim C.T., Goh J.C.H. (2009). Compressive fatigue and thermal compressive fatigue of hybrid resin base dental composites. IFMBE Proceedings, Proceedings of the13th International Conference on Biomedical Engineering, Singapore, 3–6 December 2008.

[29] Mair L.H., Stolarski T.A., Vowles R.W., Lloyd C.H. (1996). Wear: Mechanisms, manifestations and measurement. Rep. Workshop J. Dent..

[30] Kawano F., Ohguri T., Ichikawa T., Matsumoto N. (2001). Influence of thermal cycles in water on flexural strength of laboratory-processed composite resin. J. Oral Rehabil..

[31] Janda R., Roulet J.F., Latta M., Ruttermann S. (2007). Water sorption and solubility of contemporary resin-based filling materials. J. Biomed. Mater. Res. Part. B Appl. Biomater..

[32] Pieniak D., Walczak A., Niewczas A.M., Przystupa K. (2019). The Effect of Thermocycling on Surface Layer Properties of Light Cured Polymer Matrix Ceramic Composites (PMCCs) Used in Sliding Friction Pair. Materials.

[33] Walczak A., Pieniak D., Niewczas A.M., Gil L. (2018). Laboratory studies of the influence of thermal cycling on anti-wear properties of composites used in biotribological friction pairs. Tribologia.

[34] Pieniak D., Kordos P. (2019). Analysis of the degree of hydro-thermal fatigue damage of the surface layer of polymer-ceramic composites intended for operation in a biotribological node. Tribologia.

[35] Palaniappan S., Peumans M., Van Meerbeek B., Lambrechts P., Yan Y. (2013). Wear prediction in dental composites. Woodhead Publishing Series in Biomaterials, Bio-Tribocorrosion in Biomaterials and Medical Implants.

[36] Souza J., Bentes A., Reis K., Gavinha S., Buciumeanu M., Henriques B., Silva F.S., Gomes J.R. (2016). Abrasive and sliding wear of resin composites for dental restorations. Tribol. Int..

[37] Lambrechts P., Goovaerts K., Bharadwaj D., De Munck J., Bergmans L., Peumans M., Van Meerbeek B. (2006). Degradation of tooth structure and restorative materials: A review. Wear.

[38] Heintze S.D., Zellweger G., Zappini G. (2007). The relationship between physical parameters and wear of dental composites. Wear.

[39] Gale M.S., Darvell B.W. (1999). Thermal cycling procedures for laboratory testing of dental restorations. J. Dent..

[40] Li J., Li H., Foka A.S.L., Watts D.C. (2009). Multiple correlations of material parameters of light-cured dental composites. Dent. Mater..

[41] Mayworm C.D., Camargo S.S., Bastian F.L. (2008). Influence of artificial saliva on abrasive wear and microhardness of dental composites filled with nanoparticles. J. Dent..

[42] Marovic D., Panduric V., Tarle Z., Ristic M., Sariri K., Demoli N., Klaric E., Jankovic B., Prskalo K. (2013). Degree of conversion and microhardness of dental composite resin materials. J. Mol. Struct..

[43] Delfino C.S., Youssef M.N., Souza F.B., Braz R., Turbino M.L. (2012). Microhardness of a dental restorative composite resin containing nanoparticles polymerized with argon ion laser. Optik.

[44] Ilie N., Hilton T.J., Heintze S.D., Hickel R., Watts D., Silikas N., Stansbury J.W., Cadenaro M., Ferracane J.L. (2017). Academy of Dental Materials guidance-Resin composites: Part I-Mechanical properties. Dent. Mater..

[45] Łępicka M., Grądzka-Dahlke M., Pieniak D., Pasierbiewicz K., Niewczas A. (2017). Effect of mechanical properties of substrate and coating on wear performance of TiN- or DLC-coated 316LVM stainless steel. Wear.

[46] Łępicka M., Grądzka-Dahlke M., Pieniak D., Pasierbiewicz K., Kryńska K., Niewczas A. (2019). Tribological performance of titanium nitride coatings: A comparative study on TiN-coated stainless steel and titanium alloy. Wear.

[47] Łępicka M., Ciszewski A., Golak K., Grądzka-Dahlke M. (2019). A Comparative Study of Friction and Wear Processes of Model Metallic Biomaterials Including Registration of Friction-Induced Temperature Response of a Tribological Pair. Materials.

[48] Clelland N.L., Agarwala V., Knobloch L.A., Seghi R.R. (2001). Wear of enamel opposing low-fusing and conventional ceramic restorative materials. J. Prosthodont..

[49] Hahnel S., Schultz S., Trempler C., Ach B., Handel G., Rosentritt M. (2011). Two-body wear of dental restorative materials. J. Mech. Behav. Biomed. Mater..

[50] Ghazal M., Albashaireh Z.S., Kern M. (2008). Wear resistance of nanofilled composite resin and feldspathic ceramic artificial teeth. J. Prosthet. Dent..

[51] Paczkowska M., Selech J., Piasecki A. (2016). Effect of surface treatment on abrasive wear resistance of seeder coulter flap. Surf. Rev. Lett..

[52] Borrero-Lopez O., Guiberteau F., Zhang Y., Lawn B.R. (2019). Wear of ceramic-based dental materials. J. Mech. Behav. Biomed. Mater..

[53] Walczak M., Pieniak D., Niewczas A.M. (2014). Effect of recasting on the useful properties CoCrMoW alloy. Eksploat. Niezawodn..

[54] Pieniak D., Niewczas A.M., Walczak M., Zamościńska J. (2014). Influence of photopolymerization parameters on the mechanical properties of polymer – ceramic composites applied in the conservative dentistry. Acta Bioeng. Biomech..

[55] Tuncer S., Demirci M., Tiryaki M., Ünlü N., Uysal Ö. (2013). The Effect of a modeling resin and thermocycling on the surface hardness, roughness, and color of different resin composites. J. Esthet. Restor. Dent..

[56] Pieniak D., Niewczas A.M., Kordos P. (2012). Influence of thermal fatigue and ageing on the microhardness of polymer-ceramic composites for biomedical applications. Eksploat. Niezawodn..

[57] Souza R.O.A., Ozcan M., Michida S.M.A., de Melo R.M., Pavanelli C.A., Bottino M.A., Soares L.E.S., Martin A.A. (2010). Conversion degree of indirect resin composites and effect of thermocycling on their physical properties. J. Prosthodon..

[58] Ayatollahi M.R., Yahya M.Y., Karimzadeh A., Nikkhooyifar M., Ayob A.B. (2015). Effects of temperature change and beverage on mechanical and tribological properties of dental restorative composites. Mat. Sci. Eng. C-Mater..

[59] Mair L.H. (1992). Wear in dentistry—Current terminology. J. Dent..

[60] Mehl C., Scheibner S., Ludwig K., Kern M. (2007). Wear of composite resin veneering materials and enamel in a chewing simulator. Dent. Mater..

[61] Wang R., Bao S., Liu F., Jiang X., Zhang Q., Sun B., Zhu M. (2012). Wear behavior of light-cured resin composites with bimodal silica nanostructures as fillers. Mater. Sci. Eng. C-Mater. Biol. Appl..

[62] Turssi C.P., De Moraes Purquerio B., Serra M.C. (2003). Wear of dental resin composites: Insights into underlying processes and assessment methods—A review. J. Biomed. Mater. Res. Part. B.

[63] Pieniak D., Walczak A., Niewczas A.M. (2016). Comparative study of wear resistance of the composite with microhybrid structure and nanocomposite. Acta Mech. Autom..

[64] Ferracane J.L. (2013). Resin-based composite performance: Are there some things we can’t predict. Dent. Mater..

[65] Friedrich K., Friedrich K. (1986). Wear of reinforced polymers by different abrasive counterparts. Friction and Wear of Polymer Composites.

[66] Zwierzycki W. (1998). Prognozowanie Niezawodności Zużywających Się Elementów Maszyn.

[67] Truong V.T., Cock D.J., Padmanathan N. (1990). Fatigue crack propagation in posterior dental composites and prediction of clinical wear. J. Appl. Biomater..

[68] McKinney J.E., Wu W. (1982). Relationship between subsurface damage and wear of dental restorative composites. J. Dent. Res..

[69] Hu X., Marquis P.M., Shortall A.C. (1999). Two-body in vitro wear study of some current dental composites and amalgams. J. Prostht. Dent..

[70] Wieleba W. (2013). Bezobsługowe Łozyska Ślizgowe z Polimerów Termoplastycznych.

[71] de Souza J.A., Dolavale L.C., de Souza Camargo S.A. (2013). Wear mechanisms of dental composite restorative materials by two different in-vitro methods. Mater. Res..

[72] Pieniak D., Niewczas A. (2012). Phenomenological evaluation of fatigue cracking of dental restorations under conditions of cyclic mechanical loads. Acta Bioeng. Biomech..

